# Effect of Diet on HDL in Obesity

**DOI:** 10.3390/molecules29245955

**Published:** 2024-12-17

**Authors:** Tiziana Bacchetti, Camilla Morresi, Oriana Simonetti, Gianna Ferretti

**Affiliations:** 1 Department of Life and Environmental Sciences, Polytechnic University of Marche, 60131 Ancona, Italy; 2 Clinic of Dermatology, Department of Clinical and Molecular Sciences, Polytechnic University of Marche, 60126 Ancona, Italy; o.simonetti@univpm.it; 3 Department of Clinical Experimental Science and Odontostomatology, Research Center of Health Education and Health Promotion and Research Center of Obesity, Polytechnic University of Marche, 60126 Ancona, Italy; g.ferretti@univpm.it

**Keywords:** adipose tissue, dietary antioxidants, inflammation, lipoproteins, obesity, oxidative stress

## Abstract

Alterations of plasma lipoprotein levels and oxidative stress are frequently observed in obese patients, including low high-density lipoprotein (HDL) cholesterol (HDL-C) levels and alterations of HDL composition. Dysfunctional HDL with lower antioxidant and anti-inflammatory properties have also been demonstrated in obesity. There is increasing evidence that white adipose tissue (WAT) participates in several metabolic activities and modulates HDL-C levels and function. In obese subjects, the changes in morphology and function of adipose tissue lead to impaired regulatory function and are associated with a state of low-grade chronic inflammation, with increased release of pro-inflammatory adipokines and cytokines. These alterations may affect HDL metabolism and functions; thus, adipose tissue is considered a potential target for the prevention and treatment of obesity. A cornerstone of obesity prevention and therapy is lifestyle modification through dietary changes, which is reflected in the modulation of plasma lipoprotein metabolism. Some dietary components and metabolites directly affect the composition and structure of HDL and modulate its anti-inflammatory and vasoprotective properties. The aims of the review are to summarize the crosstalk between adipocytes and HDL dysfunction in human obesity and to highlight recent discoveries on beneficial dietary patterns as well as nutritional components on inflammation and HDL function in human obesity.

## 1. Introduction

Obesity is a multifactorial chronic disease defined by excess adipose mass and adipose tissue expansion, which occurs through adipocyte hypertrophy and hyperplasia. The role of white adipose tissue (WAT) in molecular mechanisms of obesity has been reviewed [1,2,3]. In addition to WAT, the human body also contains brown adipose tissue (BAT) and beige adipose tissue. The physio-pathological role of BAT has mainly been investigated in animal models. However, some recent studies have demonstrated active brown adipose tissue in adult humans, and a potential role in molecular mechanisms of dysmetabolic diseases has been proposed [4,5,6,7,8,9]. Several factors, such as poor dietary habits, sedentary lifestyle, decreased sleep duration, poor sleep quality, socioeconomic influences, and genetic factors are involved in the development of human obesity [10,11,12,13,14]. Previous epidemiological studies have demonstrated that obesity is considered a risk factor for several complications and metabolic disorders, including cardiovascular complications, type 2 diabetes, cancer, and hepatic and renal dysfunction [15,16]. Moreover, elevated body weight can influence various aspects of reproduction and fertility [17]. Therefore, an excess amount of body fat not only reduces the quality of life but also increases healthcare-associated costs.

Oxidative stress and WAT dysfunction are involved in the molecular mechanisms of the health risks of obesity. A higher generation of intracellular reactive oxygen species (ROS) and an imbalance of pro-oxidant/antioxidant enzymes has been described in human obesity as recently reviewed [18,19,20,21]. Oxidative stress is confirmed by a significant increase in biochemical markers of lipid and protein oxidation in plasma or serum lipoproteins of obese subjects [22,23,24,25]. The pro-oxidant state is linked to the excess accumulation of abdominal fat. In fact, the generation of O_2_^•−^ has been related to the increase in plasma free fatty acids (FFA) as well as excessive fat storage in WAT. In addition, FFA stimulates the production of reactive intermediates through Protein Kinase C (PKC)-dependent activation of NADPH oxidase (NOX) in cultured vascular cells [20]. The primary function of WAT is to store triglycerides as an energy reservoir; however, WAT also participates in several metabolic activities and modulates cholesterol metabolism and HDL-C levels. The higher plasma levels of triglyceride (TG), total cholesterol, cholesterol associated with low-density lipoprotein (LDL-C), and the lower levels of HDL-C levels are involved in the connection between obesity and aforementioned dysmetabolic diseases. In fact, lipoproteins, in addition to the transport of plasma lipids, exert several physio-pathological roles. Both LDL and HDL modulate membrane lipid composition and cell signaling and affect cell proliferation and apoptosis [26,27,28]. In addition, several studies have demonstrated that WAT secretes several bioactive molecules, such as cytokines and adipokines, that exert different roles and modulate systemic energy balance by regulating appetite signals from the central nerve system as well as metabolic activity in peripheral tissues [1,2,3,29,30]. Studies in human subjects and animal models have demonstrated that the endocrine function of WAT is perturbed by an excess of fat and causes dysregulated expression of several factors. Changes in the number and size of adipocytes are associated with alterations in adipokine secretion, fatty acid fluxes, and alterations in plasma lipoprotein levels. Abdominal adiposity is also involved in the development of a state of low-grade chronic inflammation. Besides WAT, the liver is an important organ involved in inflammation in human obesity. Infiltration of neutrophils and macrophages into liver and adipose tissue is involved in the activation of inflammatory pathways that are responsible for the attraction and activation of immune cells. Adipose tissue infiltrating macrophages contribute to the increased secretion of proinflammatory cytokines (i.e., TNF-α, IL-6, IL-1). A pro-inflammatory condition in human obesity is supported by the higher myeloperoxidase (MPO) activity from neutrophils isolated from obese subjects compared with lean subjects [31,32,33]. The enzyme MPO is stocked in neutrophil granules and released upon exocytosis and is one of the main factors involved in the oxidative stress of plasma lipoproteins in dysmetabolic diseases. Therefore, obesity may be viewed as a metabolic as well as an inflammatory disease [34].

Dietary factors can trigger a dynamic remodeling of adipocytes, including quantitative and qualitative alterations in adipose tissue-resident cells. Identification of biochemical factors and pro-inflammatory factors that cause dysfunctional adipose tissue could be a useful tool in the clinical setting to prevent cardiometabolic risk independently from adiposity. The alterations of WAT in obesity and the cross-link with HDL metabolism and dysfunction have been previously reviewed [35,36,37,38,39], and it has been suggested that WAT could be considered a potential target for the prevention and treatment of metabolic alterations in obesity. Over the last decade, an increasing number of studies have found that activating brown adipose tissue and browning of white adipose tissue can protect against obesity and obesity-related metabolic disease. Therefore, brown adipose tissue has gradually become an appealing therapeutic target for the prevention and reversal of obesity [4,5,6,7,8]. In this review, more recent data on the crosstalk between WAT, inflammation, and HDL in obesity have been included, with a focus on the new insights into the potential mechanisms underlying obesity, inflammation, and HDL dysfunction. Recent data on BAT and its physio-pathological role have also been summarized. In addition, we aim to highlight recent discoveries on beneficial dietary patterns as well as nutritional components and their effects on adipose tissue, inflammation, and HDL function in human obesity.

## 2. Adipose Tissue and HDL Metabolism

### 2.1. HDL Metabolism

**Figure 1:**
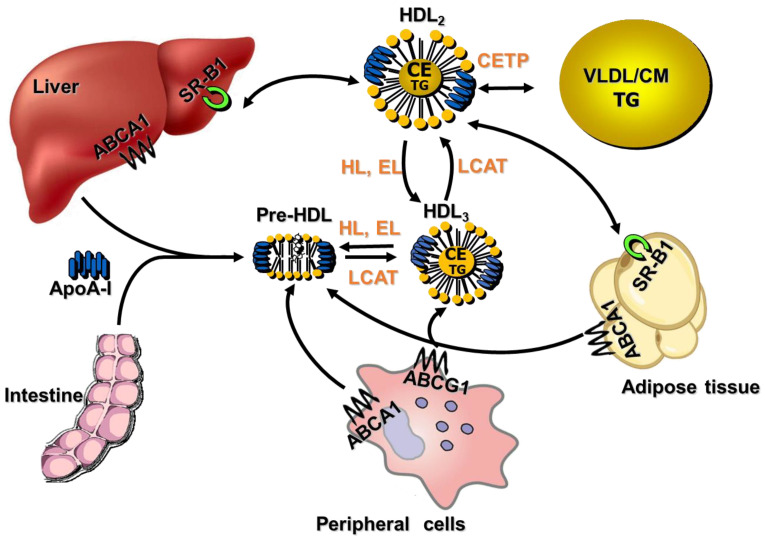
HDL biosynthesis and maturation. Biosynthesis of HDL starts with the production and secretion of apolipoprotein A-I (ApoA-I) by the liver and intestine. Lipid-poor ApoA-I interacts with ATP-binding cassette A1 (ABCA1) to acquire lipids from peripheral cells, including adipose tissue, resulting in nascent HDL (Pre-HDL). The enzyme lecithin cholesterol acyl transferase (LCAT) esterifies free cholesterol to esterified cholesterol (CE). CE migrates to the core of the discoidal HDL, promoting its maturation into spherical HDL. HDL_2_ and HDL_3_, the two subclasses of HDL, can interconvert. Cholesterol is delivered to the liver via scavenger receptor BI (SR-BI) or transferred to very low-density lipoproteins (VLDL) by cholesteryl ester transfer protein (CETP). CETP also contributes to exchanges of TG and CE between HDL and other lipoproteins. HDL-associated triglycerides and phospholipids are mainly hydrolyzed by endothelial lipase (EL) and hepatic lipase (HL). The final step of reverse cholesterol transport involves the selective uptake of CE from HDL particles by the hepatic scavenger receptor class B (SR-BI). A as bidirectional transport of cholesterol-facilitated SR-B1 has also been observed in adipose tissue.

The structure of HDL is like all lipoproteins. The surface of mature HDL is a monolayer of glycero-phospholipids, sphingolipids, and free cholesterol. At the surface, there are ApoA-I and ApoA-II, the main apolipoproteins of HDL. ApoA-I is about 70% of the protein content associated with HDL. Several other apoproteins contribute to diverse functions, including lipid transport, immune response, antioxidant properties, and hemostasis. The hydrophobic nucleus contains triglycerides (TG) and esterified cholesterol (CE). In human plasma, HDL is a highly heterogeneous population of particles ranging in size and density. Using ultracentrifugation, human HDL can be separated into two main subclasses on the basis of density: HDL_2_ (1.063–1.125 g/mL) and HDL_3_ (1.125–1.21 g/mL). The biogenesis and maturation of HDL involve several factors, membrane-bound proteins and plasma biomolecules. The liver and intestine exert a key role in HDL biogenesis. In fact, hepatocytes and intestinal cells synthesize ApoA-I, the main HDL apoprotein. Lipid-free ApoA-I acquires cholesterol and phospholipids from peripheral cells via ATP-binding cassette transporter A1 (ABCA1), forming nascent discoidal HDL particles (Figure 1). Among plasma enzymes involved in HDL remodeling and maturation, there is lecithin-cholesterol-acyl transferase (LCAT). The esterification of free cholesterol by the enzyme LCAT converts the discoidal HDL to spherical HDL particles (Figure 1). In fact, CE is more hydrophobic and migrates to the core of the discoidal HDL, promoting its maturation into spherical HDL. The increase in HDL particle size provides substrates for cholesteryl ester transfer protein (CETP). CETP contributes to exchanges of TG and CE between HDL and other lipoproteins (Figure 1). Among the enzymes involved in HDL metabolism there are hepatic lipase (HL) and endothelial lipase (EL). HL not only hydrolyzes triglyceride and phospholipid in HDL but also stimulates HDL uptake of CE by hepatocytes [40]. EL is a member of the lipase family; the enzyme behaves as phospholipase and also contributes to the hydrolysis of triglyceride to a lesser extent. Recent studies have suggested that EL is an important modulator of HDL concentrations [41] (Figure 1).

HDL exerts several key roles. One of the main functions of HDL is the ability to promote reverse cholesterol transport, the uptake of excess cholesterol from peripheral cells, and the transport to the liver for excretion. This process is considered the major antiatherogenic effect of HDL and has emerged as a better predictor of cardiovascular (CV) risk compared to merely plasma HDL cholesterol (HDL-C) levels [42]. Antioxidant and anti-inflammatory properties of HDL have been widely described [26,28]. In the next paragraph, HDL alterations observed in human obesity and potential mechanisms and the role of WAT and BAT are described.

### 2.2. Role of WAT

WAT exerts a key role in the metabolism of plasma lipids and lipoproteins. In fact, apart from storing TG, WAT is also the major store for cholesterol within the body and, therefore, represents a large pool of substrate to support HDL biogenesis. Over half of the total body cholesterol, about 1–2 mg of cholesterol per gram of wet weight, may reside within this tissue. Low levels of HDL-C have been frequently observed in obese subjects. These alterations can be related to changes in the morphology and function of adipose tissue [35,36,37,38,39]. Table 1 summarizes biochemical factors expressed on WAT and their modulatory roles on HDL composition and metabolism.

The relationship between WAT, lipoproteins, and HDL-C levels and metabolism is supported by the expression of membrane receptors for LDL and membrane cholesterol transporters such as ABCA1 and scavenger receptor class B type I (SR-BI) on adipocytes [39,43,44]. Cholesterol influx in adipocytes is mediated by interactions between LDL particles with LDL receptors. Adipocytes may also obtain cholesterol from the interaction of their SR-B1 receptors with hepatic/intestinal HDL particles. Cholesterol efflux by adipocytes involves the ABCA1 transporter, as well as bidirectional facilitated SR-B1 transport, both of which may provide a gateway for cholesterol efflux to ApoA-I or HDL particles (Figure 1).

**Figure 2:**
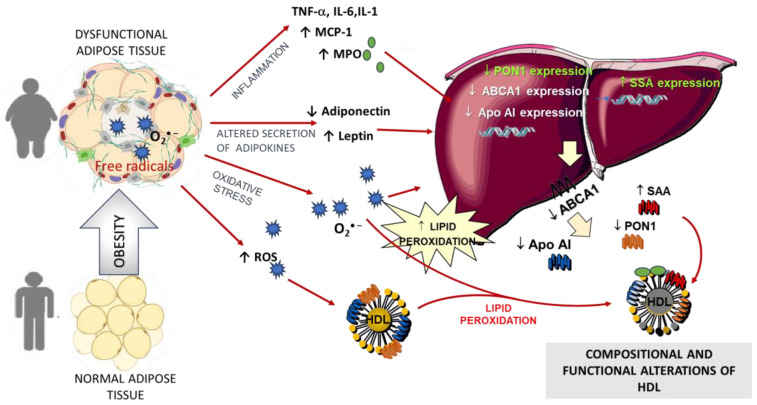
Cross-talk between adipocytes and HDL in obesity. In obesity, altered secretion of adipokines by adipose tissue modulates gene expression of liver proteins. ApoA-I and ABCA1 are downregulated; thus, HDL assembly is decreased in the liver. Moreover, hypertrophic adipocytes also release inflammatory molecules and ROS, which are able to decrease hepatic ApoA-I and PON1 expression and secretion. The higher level of SAA displaces PON1 and ApoA-I on the HDL surface. ROS may induce oxidative damage in hepatic cells and lipid peroxidation of HDL. All these events lead to lower HDL levels and formation of HDL with altered composition and functions. ABCA1, ATP-binding cassette transporter A1; ApoA-I, apolipoprotein A-I; HDL, high-density lipoproteins; IL-1, interleukin-1; IL-6, interleukin-6; MCP-1, monocyte chemoattractant protein-1, MPO, myeloperoxidase; PON1, paraoxonase-1; SAA, serum amyloid A; TNF-α, tumor necrosis factor-α. ↓ decrease; ↑ increase.

During inflammation, a downregulation of ABCA1 and SR-BI expression impairs cholesterol efflux from adipocytes to HDL [39,45]. These data confirm the impact of adipose tissue on the modulation of HDL-C levels. Figure 2 summarizes the metabolic alterations of WAT in obesity. Adipose tissue is also one of the major sources of CETP expression [46]. CETP activity modulates lipid exchange between HDL and different lipoprotein fractions. In addition, an increased CETP-mediated transfer of TG on HDL was reported in obesity [47]. These alterations may contribute to lipid compositional changes with an increase in the TG content of HDL and a depletion of CEs and PLs. Therefore, an increased CETP-mediated transfer of TG on HDL and the subsequent higher hydrolysis of TG-enriched HDL by lipase appears to be the mechanism that could underline the lower HDL-C levels and contribute to modifications of HDL heterogeneity with a shift of large HDL to small and dense HDL particles [35]. The lipid compositional alterations (reduced levels of free cholesterol, cholesteryl ester, and phospholipids and higher levels of TG and products of lipid peroxidation) observed in HDL of human subjects, and in particular, the increased ratio TG/CE ratio, modulates the physico-chemical properties of HDL hydrophobic core. Lipids exert a conformational role on HDL apoprotein and modulate alterations that reflect modifications of lipid-apoprotein interactions. These alterations can explain the impaired HDL functionality in obese subjects, including decreased HDL-induced eNOS activation [48], higher HDL susceptibility to oxidation [22,23], and a decrease in their antioxidant activity [23,24,35,49,50] (Table 2). The lipid and apoprotein compositional alterations of HDL, including the TG enrichment and the decrease in ApoA-I, are also associated with a decrease in the vasorelaxant effect of HDL [50]. A decreased capacity of HDL isolated from obese subjects to promote cholesterol efflux from fibroblasts was reported by Sasahara et al. [35,51], and a significant inverse correlation between cholesterol efflux capacity and the body mass index (BMI) has been shown [51]. In addition, HDL isolated from obese patients exhibits a lower ability to protect endothelial cells against oxidized LDL (ox-LDL) [50]. The lower activity of the enzyme paraoxonase 1 (PON1) in HDL of obese subjects may be related to a pro-inflammatory state and/or to compositional changes in HDL [22,23,24]. Other enzymes modulate HDL levels and HDL heterogeneity in obesity, including the activity of hepatic lipase (HL) and lipoprotein lipase (LPL). HL activity is increased in obesity, leading to faster clearance of triglyceride-rich HDL [52,53]. Moreover, TG-enriched HDL, as those observed in obesity, is a more susceptible substrate for HL and, therefore, undergoes rapid hydrolysis [54].

Fat accumulation and low-grade inflammation in WAT cause a dysregulated production of adipokines, leading to a reduction of serum levels of adiponectin [56]. Adiponectin, an anti-inflammatory and anti-atherogenic adipokine secreted from WAT [56] acts as a key mediator of the crosstalk between adipose tissue, the liver, and the vascular wall [56,57]. The adiponectin levels indicate an increase in low-grade systemic inflammation [57]. Plasma adiponectin levels in humans account for 0.01% of the total human plasma proteins, and this makes it the most abundant adipose tissue protein [58]. Plasma levels of adiponectin are significantly lower in obese subjects [59], those with metabolic syndrome [60], and type 2 diabetic patients [61]. Previous studies have shown that serum levels of adiponectin are positively correlated with the serum level of HDL-C [62]. In particular, HDL-C is positively correlated with high-molecular-weight (HMW) adiponectin, which is considered the most biologically active fraction of adiponectin [63]. Other authors have confirmed that dysfunction of adipose tissue due to elevated insulin resistance index and low values of adiponectin are associated with a higher probability of low HDL-C and small HDL [64]. The study of the mechanism by which adiponectin affects HDL-C levels has demonstrated that the molecule increases mRNA expression and protein secretion of ApoA-I from HepG2 cells and upregulates ABCA1 expression. Therefore, it has been suggested that adiponectin might increase hepatic HDL assembly [65].

Obesity is also associated with an imbalance of leptin. Elevated levels of leptin are observed in overweight and obese individuals [66]. In obesity, leptin is not able to adequately regulate energy expenditure. Obese individuals with low basal metabolic rates, despite high circulating leptin concentrations, are commonly referred to as leptin-resistant [66]. Both in obese children and adults, plasma leptin levels are negatively correlated with HDL-C [35,67]. Leptin has been reported to modulate the regulator of hepatic SR-BI expression and, thus, HDL cholesterol levels [68], however, the molecular mechanisms have not been clarified [69,70,71]. Recent studies have demonstrated that CETP levels are correlated with leptin levels. Therefore, the obesity-associated increase in CETP production is thought to affect HDL-C levels [72,73].

As aforementioned, human obesity is considered a chronic low-grade inflammatory disease. When hypertrophic adipocytes predominate, their size correlates with increased inflammatory adipokine expression and secretion, including leptin, IL-6, IL-8, TNF-α, IL-1β, chemokines such as monocyte chemotactic protein-1 (MCP-1), and enzymes such as MPO [74]. The inflammatory cytokines stimulate the production of acute-phase proteins, such as C-reactive protein (CRP), fibrinogen, and serum amyloid A (SAA). MCP-1 increases the infiltration of macrophages and lymphocytes, exacerbating proinflammatory conditions. TNF-α expression is also elevated in the adipose tissue of experimental models of obesity. The study of the effect of the secretion of pro-inflammatory molecules in obesity demonstrated correlations with alterations of HDL structure and functions. For instance, TNF-α and IL-1β reduce hepatic ApoA-I gene expression in a dose-dependent manner [75] (Figure 2). This effect occurs at the transcriptional level through a reduction in ApoA-I promoter activity and is mediated through extracellular-signal-regulated kinase and c-jun-N-terminal kinase signaling and a cytokine-responsive element within site A of the promoter [76]. Alterations in hepatic PON1 synthesis, secretion, stability, and its association with HDL or direct inactivation of the PON1 can also be related to a pro-inflammatory state. Both cell membrane receptor-mediated signaling pathways and nuclear receptors (such as peroxisome proliferator-activated receptors, PPARs) modulate HDL synthesis and gene expression of PON1 [77]. Among modulating factors of hepatic synthesis of PON1, there are reactive oxygen species (ROS) and lipid peroxidation products [77,78]. The decrease in PON1 activity and the increased level of markers of lipid peroxidation in the blood of obese subjects could be due to the increased production of ROS and pro-inflammatory cytokines (TNF-α, IL-1, and IL-6) from adipose tissue and liver [23,24]. Previous studies have demonstrated that PON1 is a lipid-dependent enzyme and that HDL modulates PON1 secretion by the liver, the enzyme stability, and stimulates its activity [79]. Dietary lipids induce changes in HDL lipid composition and physico-chemical properties [78]. Since HDL has a mean biological half-life longer with respect to other lipoproteins (about four-six days), it has been suggested that dietary lipids could induce compositional changes in HDL lipids and modifications of HDL liver secretion and binding of PON1 to the surface of HDL. In addition, it has been demonstrated that the fatty acid composition of HDL modulates its sensibility to lipid peroxidation. HDL oxidized in vitro behaves as dysfunctional HDL with a reduced capacity to stabilize/improve the activity of secreted PON1 [80]. A low-grade inflammation state in obesity is also supported by the increased levels of serum amyloid A (SAA) [35]. The hepatic production of SAA, one of the acute phase proteins, is related to the inflammatory state of the obese and to the elevated plasma levels of pro-inflammatory cytokines such as IL-6, which modulate the synthesis of hepatic proteins. The association between SAA and obesity has been recently reviewed [55,81]. Overproduction of SAA has also been observed in adipose tissue of obese subjects, particularly in morbidly obese subjects. In detail, SAA is strictly related to the HDL3 subclass [82]. The increased levels of SAA observed during the acute phase response displace ApoA-I at the HDL surface. These compositional modifications exert a negative effect on ABCA1-mediated lipidation of ApoA-I, with reduced formation of nascent HDL [83] and decreased PON1 levels [78] (Figure 2). The ApoA-I displacement is associated with decreased activities of LCAT and PON1 on the HDL surface [35,84]. In fact, ApoA-I exerts a modulatory role in the activities of the enzymes LCAT and PON1. Therefore, SAA-rich HDL are described as dysfunctional, and their ability to regulate cholesterol efflux capacity is impaired [85]. In addition, SAA-enriched HDL is implicated in the decreased HDL levels observed during inflammation [84]. Moreover, SAA is known to increase the production of cytokines, ROS, and nitric oxide and to contribute to the molecular mechanisms involved in the development of complications associated with obesity, including insulin resistance.

Other factors secreted by the liver or adipose tissue can affect the HDL functions of obese subjects. In fact, the pro-inflammatory state in obesity is also related to the activation of inflammatory pathways that are responsible for the attraction and activation of immune cells both in the liver and adipose tissue. Among molecules released after the recruitment and activation of phagocytes, there is the enzyme myeloperoxidase (MPO). An increased attention is devoted to MPO and its physio-pathological relevance. In fact, MPO is a heme peroxidase considered a principal enzyme in the innate immune response, and it is discharged into the extracellular compartment following phagocyte activation [86]. The enzyme uses the substrates hydrogen peroxide (H_2_O_2_) and nitric oxide (NO) to produce nitrated reactive species; these molecules trigger oxidative damage, especially on HDL and LDL. In a proinflammatory condition, Apo A-I can be damaged by MPO [87]. Higher levels of MPO are commonly found in obese adults [31,32,88,89,90,91]. Adipose and muscle tissue did not contain increased numbers of MPO-expressing cells in severely obese individuals. Therefore, it has been proposed that circulating neutrophils are activated to a greater extent in severely obese subjects, and a chronic inflammatory condition is associated with morbid obesity. MPO-dependent modifications of HDL have been claimed as possible pathophysiological mechanisms for higher risk for atherosclerosis. In fact, high nitration levels in ApoA-I tyrosine residues were found in obese women and associated with lower cholesterol efflux of nitrated HDL [92]. Some authors reported that MPO and PON1 correlated with each other, as well as with markers of oxidative stress (asymmetric dimethyl arginine) and inflammation (soluble CD40 ligand and soluble intracellular adhesion molecule) [93]. In a similar population of overweight dyslipidemic patients, MPO correlated with suppressed PON1 arylesterase activity and the occurrence of vascular complications [94].

### 2.3. Role of BAT

The role of BAT in obesity has been paid more recently with respect to WAT. BAT content declines in adults and accounts for 0.05–0.1% of body weight in adults. The physio-pathological role of BAT has been mainly investigated in animal models, and the modulatory role of BAT on metabolic and cardiovascular disease in humans is still debated. However, some recent studies have demonstrated active brown adipose tissue in adult humans [95]. It has been demonstrated that human subjects with BAT activity have lower plasma triglycerides and higher HDL cholesterol [6,8,9]. Cholesterol-enriched chylomicron remnants and HDL generated by intravascular lipolysis in BAT are cleared more rapidly by the liver, explaining the potential antiatherogenic effects of BAT activation [96]. The interest in BAT in inflammation and obesity is supported by proteomics analysis, which also provided data on some cytokines expressed by BAT (frontiers), including leptin, adiponectin, and IL6 [97,98].

## 3. Dietary Anti-Inflammatory and Antioxidant Strategies in Human Obesity

**Figure 3:**
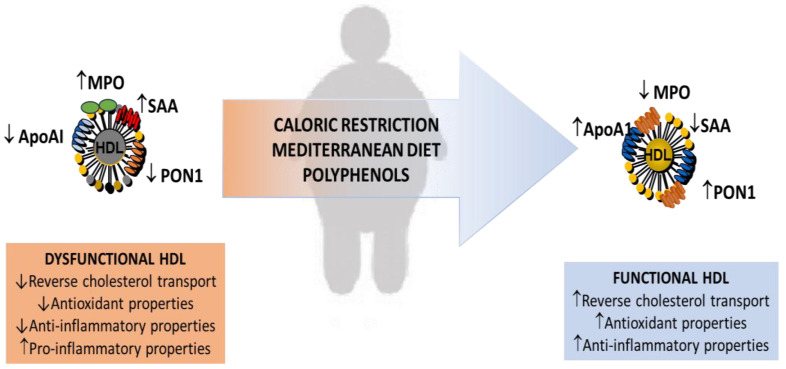
Effect of dietary factors on HDL functionality in obesity. Caloric restriction, Mediterranean diet, and antioxidation intake, including polyphenols, are associated with a reduction in pro-inflammatory cytokines and markers of oxidative stress with subsequent beneficial effects in terms of HDL functionality in human obesity. ApoA-I, apolipoprotein A-I; HDL, high-density lipoproteins; MPO, myeloperoxidase; PON1, paraoxonase-1; SAA, serum amyloid A. ↓ decrease; ↑ increase.

Several strategies have been proposed to prevent and treat human obesity. Dietary treatment and changes in lifestyle are still the major therapeutic bases. The literature data report that different dietary treatments and/or nutritional factors affect body weight and lipoprotein levels in human subjects in normal and pathological conditions [99,100]. A thorough discussion of dietary and nutritional therapies is beyond the scope of this review and reviewed elsewhere [101]. In addition, plasma lipid and lipoprotein levels and effects of nutritional intake may vary depending on genetic factors, age, race, and environmental factors. Gender differences are also important, as well as the dynamics of weight changes [102]. Here, we summarize some recent studies that demonstrate that the effects of some dietary treatments on weight reduction are associated with an improvement in the levels of inflammatory markers and a decrease in levels of oxidative stress. For instance, human studies on caloric restriction in non-obese and obese participants have shown that even short-term dietary interventions with moderate caloric restriction cause a reduction in body weight as well as an improvement of biochemical parameters and cardiometabolic health parameters [103,104,105]. The beneficial effects include a decrease in levels of markers of oxidative stress and a decrease in fasting insulin levels as well as lower circulating levels of cytokine such as TNF-α [106] (Figure 3). The potential anti-inflammatory effect of caloric restriction in morbidly obese patients has been demonstrated by recent studies. Lopez-Domenech et al. have shown that a very low-calorie diet for six months improved anthropometric and biochemical parameters and improved the inflammatory response with a decrease in the activity of the pro-oxidant enzyme MPO and increased antioxidant capacity such as catalase activity [107]. Kanikowska et al. have also demonstrated that energy restriction improved oxidative stress markers in obese patients with a decrease in MPO and serum oxidative stress markers [108]. Moreover, a decrease in systemic inflammatory markers was shown after weight loss with lower concentrations of CRP, IL-6, and TNF-α after dietary therapy. A decrease in leptin, a pro-inflammatory and pro-oxidative activity factor, has also been observed after dietary intervention [108]. Modifications of the levels of biochemical markers of inflammation have been described by Montefusco et al. after a 6-month hypocaloric diet in patients with metabolic syndrome [109]. The decrease in pro-inflammatory cytokine levels was associated with lipid compositional changes in HDL [109]. Weight loss produced by calorie restriction in obese subjects is also associated with a decrease in levels of SAA mRNA and protein [110] and with increases in plasma levels of adiponectin [111]. A recent study has confirmed that weight loss produced a significant decrease in oxidative stress, an increased expression of antioxidant enzymes, and a decrease in the levels of inflammatory markers, including hs-CRP [112]. All the aforementioned studies confirm the connection between obesity and pro-inflammatory state and the positive effect of caloric restriction on weight loss and pro-inflammatory pathways. Anti-inflammatory and protective effects against oxidative stress are also related to eating habits and patterns, including the Mediterranean diet (MedDiet) associated with or without physical exercise. The role of MedDiet in the management and prevention of obesity has been recently reviewed, and a greater reduction of body weight has been described with respect to other dietary treatments [113]. Moreover, subjects with a higher adherence to MedDiet had a lower risk of gaining weight over time. As far as concerns the effect of MedDiet on inflammatory markers, an improvement of HDL function due to suppression of MPO-mediated oxidative stress with a decrease in the levels of 3-chlorotyrosine and 3-nitrotyrosine has been demonstrated in HDL after 12 weeks of the Mediterranean diet and exercise [114]. The compositional modifications were associated with an improvement in HDL cholesterol efflux capacity [114] (Figure 3). Among the nutritional factors involved in the protective effects of MedDiet, it has been proposed that a key role is exerted by fruit and vegetables’ bioactive components. Several studies, mainly carried out in animal models and in cell culture, have demonstrated the anti-inflammatory and antioxidant properties of bioactive molecules such as polyphenols and carotenoids contained in vegetable foods [115,116,117]. The anti-obesity effects of polyphenol-rich diets have been related to different mechanisms, such as the ability of polyphenols to interact, directly or indirectly, with adipose tissues (preadipocytes, adipose stem cells, and immune cells) and modulate their behavior [118,119]. Recent data confirm the beneficial effects of vegetable food components and further support the dietary recommendations that emphasize diets rich in fruits and vegetables for the prevention of chronic diseases associated with oxidative stress and inflammation [117,120,121], including obesity [119,122,123]. Among polyphenols, some recent clinical trials have investigated the effect of foods rich in anthocyanins (including tart cherries and strawberry beverages) on inflammation associated with obesity [121]. A randomized, double-blinded clinical study showed a reduction in abdominal fat, TG, and LDL levels, with decreasing TNF-α and MCP-1 levels [124]. Contrasting data have been obtained by other studies. Zunino et al. showed that people consuming 80 g/serving of freeze-dried strawberry powder mixed with food and drinks for 3 weeks had no effect on inflammatory markers (IL-6, IL-1β, TNF-α) [125]. Similarly, consumption of commercially available red orange juice (250 mg anthocyanins/day) for 12 weeks did not show any effect on body weight or plasma inflammatory markers [126]. Possible reasons for the lack of beneficial effects of anthocyanins on obesity-induced inflammation in these studies are the use of relatively low dosages and the short length of the intervention. Fewer studies have investigated the effects of caloric restriction and intake of bioactive compounds on the composition and function of HDL of overweight or obese subjects (Figure 3). Increased plasma levels of ApoA-I and a decrease in the levels of CETP in the absence of modifications of the cholesterol efflux capacity of HDL from THP-1 cells have been observed in diabetic and obese patients treated with a low-calorie diet (for sixteen weeks) [127]. Another study has shown that weight loss was associated with an increased activity of the enzyme PON1 after a three-month treatment, including caloric restriction and physical activity in obese patients with metabolic syndrome [128] (Figure 3). Other authors have not confirmed the effect of a low-calorie diet on PON1 activity [129]. More recent studies have shown an effect of polyphenol intake on HDL composition and functional parameters, such as PON1 activity and cholesterol efflux capacity [130,131] (Figure 3). The study of Predimed found an association between the intake of polyphenol subclasses except phenolic acids and lignans and higher HDL-c levels in a MetS population of overweight or obese adults. Higher intake of all the subclasses of polyphenols was associated with a better profile of the components of MetS, especially with HDL-C levels [132]. Similar findings were described in the HELENA study [133], where flavonoid intake was associated with lower BMI. Research on the mechanisms of action involved in the anti-obesogenic properties of flavonoids suggests that the improvements in glucose homeostasis are promoted by reducing insulin resistance and decreasing oxidative stress levels [134]. Intake of anthocyanin-rich blueberries over a 6-month period resulted in increased HDL-C levels, as well as HDL particle number and improved vascular function in overweight and obese subjects [135]. The effect of green tea on obese animals has been recently reviewed [136]. Green tea appears to act as a protective agent for dyslipidemia in obesity-induced animals. Supplementation with green tea extract containing 208 mg epigallocatechin gallate (EGCG) for twelve weeks reduced total cholesterol, triglycerides, low-density lipoprotein cholesterol, and increased HDL-C [137]. Studies in overweight and obese women have demonstrated a significant increase in HDL-C following intake of green as reviewed [138]. Further studies will be necessary to better investigate the bioavailability of polyphenols and their kinetics of absorption to find the best combination of products to be used in nutritional therapy. Well-controlled long-term studies showing the effects of these dietary interventions in humans have not been fully explored. The improvements are not always maintained over time, and many subjects have difficulty maintaining healthy behavior changes, many participants regain half of the lost weight after dietary treatments within a year and return to baseline weight within 3–5 years [139]. Therefore, long-term maintenance of lost weight is the primary challenge of obesity treatment. Different strategies can contribute to long-term weight maintenance, including specific counseling after the treatment. Post-dietary treatments should be organized to motivate participants, improve their nutritional knowledge of the effects of correct food choices on health, and make them understand the importance of lifestyle in the prevention of chronic diseases.

## 4. Conclusions

Human obesity is associated with oxidative stress, inflammation, and alterations of HDL-C levels and functions. Dysfunctional hypertrophic adipose tissue in obesity exhibits a disturbed secretory pattern associated with increased secretion of pro-inflammatory cytokines and adipokines. These events promote alterations of the synthesis of ApoA-I and other molecules involved in cholesterol transport and functionality of HDL. Dysfunctional HDL with lower antioxidant and anti-inflammatory properties, as those observed in obese human subjects, may be involved in the development of several complications associated with obesity, including cardiovascular complications, type 2 diabetes, cancer, and hepatic and renal dysfunction, so they represent a possible therapeutic target. Dietary factors can trigger a dynamic remodeling of adipocytes of WAT with consequences in the reduction of inflammation and oxidative stress and beneficial effects in terms of HDL levels and functionality. Among dietary treatments, MedDiet exerts a protective role in the management and prevention of obesity, and a greater reduction of body weight has been described with respect to other dietary treatments. Further studies are needed to draw firm conclusions on the effects of bioactive phytonutrients and to better investigate the effect of dietary strategies, caloric restriction on body weight, and the molecular mechanisms involved in the cross-talk between adipose tissue and HDL in human obesity. It also has to be taken into account that different factors modulate the success of dietary interventions, including the motivation and willingness of participants and their adherence to the prescription or provided diet. Studies investigating short-term calorie restriction in human subjects demonstrate improvement in biochemical markers, weight, and decreases in markers of oxidative stress and inflammation. However, well-controlled long-term studies showing the effects of these dietary interventions in humans have not been fully explored. The improvements are not always maintained over time, and many subjects have difficulty maintaining healthy behavior changes, many participants regain half of the lost weight within a year and return to baseline weight within 3–5 years. Therefore, long-term maintenance of lost weight is the primary challenge of obesity treatment. Different strategies can contribute to long-term weight maintenance, including specific counseling after the treatment. Post-dietary treatments should be organized to motivate participants, improve their nutritional knowledge of the effects of correct food choices on health, and make them understand the importance of lifestyle in the prevention of chronic diseases.

## Figures and Tables

**Table 1:** Adipocyte proteins and their modulatory role of HDL cholesterol and HDL function.

Factors Produced by Adipocytes	Role in HDL Metabolism
ATP-binding cassette A1/G1 (ABCA1/G1)	Regulation of the efflux of intracellular cholesterol to HDL
Scavenger receptor class B type I (SR-BI)	Promotion of the cholesterol transport from adipocytes to HDL Bidirectional flux of cholesterol
Cholesteryl ester-transfer protein (CETP)	Transfer of CE from HDL to VLDL and LDL and the transfer of TG from these particles to HDL
Serum amyloid protein (SAA)	Pro-inflammatory role
Tumor necrosis factor (TNF-α)	Pro-inflammatory role
Adiponectin	Activation LPL; modulation of hepatic production of Apo-AI and ABCA1, anti-inflammatory role
Leptin	Regulation of hepatic SR-BI expression and HDL cholesterol levels
Interleukins (IL) (IL-1β and IL-6)	Pro-inflammatory role

**Table 2:** Alterations of HDL composition and functions in human obese subjects. ↓ decrease; ↑ increase.

Alteration of HDL Composition and Functions	References
Composition	
↓ levels of ApoA-I, free cholesterol, cholesteryl ester and phospholipids	[35]
↑ Triglycerides	[35]
↑ SAA content	[55]
Functions	
↓ PON1 activity	[23,24]
↑ LCAT	[35]
↓ capacity to promote cholesterol efflux from fibroblasts	[51]
↓ ability to protect endothelial cells against Ox-LDL	[50]
↓ eNOS activation	[48]
↑ susceptibility to oxidation	[23,24]
↓ vasorelaxant effect	[50]

## Data Availability

No new data were created or analyzed in this study.

## References

[1] Carobbio S., Pellegrinelli V., Vidal-Puig A. (2024). Adipose Tissue Dysfunction Determines Lipotoxicity and Triggers the Metabolic Syndrome: Current Challenges and Clinical Perspectives. Adv. Exp. Med. Biol..

[2] White U. (2023). Adipose tissue expansion in obesity, health, and disease. Front. Cell Dev. Biol..

[3] Reyes-Farias M., Fos-Domenech J., Serra D., Herrero L., Sanchez-Infantes D. (2021). White adipose tissue dysfunction in obesity and aging. Biochem. Pharmacol..

[4] Negroiu C.E., Tudorascu I., Bezna C.M., Godeanu S., Diaconu M., Danoiu R., Danoiu S. (2024). Beyond the Cold: Activating Brown Adipose Tissue as an Approach to Combat Obesity. J. Clin. Med..

[5] Prapaharan B., Lea M., Beaudry J.L. (2024). Weighing in on the role of brown adipose tissue for treatment of obesity. J. Pharm. Pharm. Sci..

[6] Greenhill C. (2024). The role of brown adipose tissue in metabolic health. Nat. Rev. Endocrinol..

[7] Ziqubu K., Dludla P.V., Mthembu S.X.H., Nkambule B.B., Mabhida S.E., Jack B.U., Nyambuya T.M., Mazibuko-Mbeje S.E. (2023). An insight into brown/beige adipose tissue whitening, a metabolic complication of obesity with the multifactorial origin. Front. Endocrinol..

[8] Becher T., Palanisamy S., Kramer D.J., Eljalby M., Marx S.J., Wibmer A.G., Butler S.D., Jiang C.S., Vaughan R., Schoder H. (2021). Brown adipose tissue is associated with cardiometabolic health. Nat. Med..

[9] Xiang A.S., Meikle P.J., Carey A.L., Kingwell B.A. (2018). Brown adipose tissue and lipid metabolism: New strategies for identification of activators and biomarkers with clinical potential. Pharmacol. Ther..

[10] Lambert D.C., Kane J., Newberry C. (2024). Lifestyle Therapy for Obesity. Gastrointest. Endosc. Clin. N. Am..

[11] Wojcik M., Alvarez-Pitti J., Koziol-Kozakowska A., Brzezinski M., Gabbianelli R., Herceg-Cavrak V., Wuhl E., Lucas I., Radovanovic D., Melk A. (2023). Psychosocial and environmental risk factors of obesity and hypertension in children and adolescents-a literature overview. Front. Cardiovasc. Med..

[12] Endalifer M.L., Diress G. (2020). Epidemiology, Predisposing Factors, Biomarkers, and Prevention Mechanism of Obesity: A Systematic Review. J. Obes..

[13] Bouchard C. (2010). Defining the genetic architecture of the predisposition to obesity: A challenging but not insurmountable task. Am. J. Clin. Nutr..

[14] Heber D. (2010). An integrative view of obesity. Am. J. Clin. Nutr..

[15] Jin X., Qiu T., Li L., Yu R., Chen X., Li C., Proud C.G., Jiang T. (2023). Pathophysiology of obesity and its associated diseases. Acta Pharm. Sin. B.

[16] Tsai A.G., Williamson D.F., Glick H.A. (2011). Direct medical cost of overweight and obesity in the USA: A quantitative systematic review. Obes. Rev..

[17] Gautam D., Purandare N., Maxwell C.V., Rosser M.L., O’Brien P., Mocanu E., McKeown C., Malhotra J., McAuliffe F.M. (2023). FIGO Committee on Impact of Pregnancy on Long-term Health; et al. The challenges of obesity for fertility: A FIGO literature review. Int. J. Gynaecol. Obstet..

[18] Balan A.I., Halatiu V.B., Scridon A. (2024). Oxidative Stress, Inflammation, and Mitochondrial Dysfunction: A Link between Obesity and Atrial Fibrillation. Antioxidants.

[19] Perez-Torres I., Castrejon-Tellez V., Soto M.E., Rubio-Ruiz M.E., Manzano-Pech L., Guarner-Lans V. (2021). Oxidative Stress, Plant Natural Antioxidants, and Obesity. Int. J. Mol. Sci..

[20] Manna P., Jain S.K. (2015). Obesity, Oxidative Stress, Adipose Tissue Dysfunction, and the Associated Health Risks: Causes and Therapeutic Strategies. Metab. Syndr. Relat. Disord..

[21] Colak E., Pap D. (2021). The role of oxidative stress in the development of obesity and obesity-related metabolic disorders. J. Med. Biochem..

[22] Ferretti G., Bacchetti T., Masciangelo S., Bicchiega V. (2010). HDL-paraoxonase and membrane lipid peroxidation: A comparison between healthy and obese subjects. Obesity.

[23] Ferretti G., Bacchetti T., Masciangelo S., Grugni G., Bicchiega V. (2012). Altered inflammation, paraoxonase-1 activity and HDL physicochemical properties in obese humans with and without Prader-Willi syndrome. Dis. Model. Mech..

[24] Ferretti G., Bacchetti T., Moroni C., Savino S., Liuzzi A., Balzola F., Bicchiega V. (2005). Paraoxonase activity in high-density lipoproteins: A comparison between healthy and obese females. J. Clin. Endocrinol. Metab..

[25] Janac J.M., Zeljkovic A., Jelic-Ivanovic Z.D., Dimitrijevic-Sreckovic V.S., Vekic J., Miljkovic M.M., Stefanovic A., Kotur-Stevuljevic J.M., Ivanisevic J.M., Spasojevic-Kalimanovska V.V. (2020). Increased Oxidized High-Density Lipoprotein/High-Density Lipoprotein-Cholesterol Ratio as a Potential Indicator of Disturbed Metabolic Health in Overweight and Obese Individuals. Lab. Med..

[26] Schoch L., Alcover S., Padro T., Ben-Aicha S., Mendieta G., Badimon L., Vilahur G. (2023). Update of HDL in atherosclerotic cardiovascular disease. Clin. Investig. Arterioscler..

[27] Negre-Salvayre A., Auge N., Camare C., Bacchetti T., Ferretti G., Salvayre R. (2017). Dual signaling evoked by oxidized LDLs in vascular cells. Free Radic. Biol. Med..

[28] Negre-Salvayre A., Dousset N., Ferretti G., Bacchetti T., Curatola G., Salvayre R. (2006). Antioxidant and cytoprotective properties of high-density lipoproteins in vascular cells. Free Radic. Biol. Med..

[29] Choe S.S., Huh J.Y., Hwang I.J., Kim J.I., Kim J.B. (2016). Adipose Tissue Remodeling: Its Role in Energy Metabolism and Metabolic Disorders. Front. Endocrinol..

[30] McArdle M.A., Finucane O.M., Connaughton R.M., McMorrow A.M., Roche H.M. (2013). Mechanisms of obesity-induced inflammation and insulin resistance: Insights into the emerging role of nutritional strategies. Front. Endocrinol..

[31] Zur B., Look M., Holdenrieder S., Stoffel-Wagner B. (2011). Elevated plasma myeloperoxidase concentration in adults with obesity. Clin. Chim. Acta.

[32] Qaddoumi M.G., Alanbaei M., Hammad M.M., Al Khairi I., Cherian P., Channanath A., Thanaraj T.A., Al-Mulla F., Abu-Farha M., Abubaker J. (2020). Investigating the Role of Myeloperoxidase and Angiopoietin-like Protein 6 in Obesity and Diabetes. Sci. Rep..

[33] Olza J., Aguilera C.M., Gil-Campos M., Leis R., Bueno G., Martinez-Jimenez M.D., Valle M., Canete R., Tojo R., Moreno L.A. (2012). Myeloperoxidase is an early biomarker of inflammation and cardiovascular risk in prepubertal obese children. Diabetes Care.

[34] Ndrepepa G. (2019). Myeloperoxidase—A bridge linking inflammation and oxidative stress with cardiovascular disease. Clin. Chim. Acta.

[35] Stadler J.T., Lackner S., Morkl S., Trakaki A., Scharnagl H., Borenich A., Wonisch W., Mangge H., Zelzer S., Meier-Allard N. (2021). Obesity Affects HDL Metabolism, Composition and Subclass Distribution. Biomedicines.

[36] Stadler J.T., Marsche G. (2020). Obesity-Related Changes in High-Density Lipoprotein Metabolism and Function. Int. J. Mol. Sci..

[37] Rashid S., Genest J. (2007). Effect of obesity on high-density lipoprotein metabolism. Obesity.

[38] Zhang T., Chen J., Tang X., Luo Q., Xu D., Yu B. (2019). Interaction between adipocytes and high-density lipoprotein:new insights into the mechanism of obesity-induced dyslipidemia and atherosclerosis. Lipids Health Dis..

[39] Zhang Y., McGillicuddy F.C., Hinkle C.C., O’Neill S., Glick J.M., Rothblat G.H., Reilly M.P. (2010). Adipocyte modulation of high-density lipoprotein cholesterol. Circulation.

[40] Thuren T. (2000). Hepatic lipase and HDL metabolism. Curr. Opin. Lipidol..

[41] Maugeais C., Tietge U.J., Broedl U.C., Marchadier D., Cain W., McCoy M.G., Lund-Katz S., Glick J.M., Rader D.J. (2003). Dose-dependent acceleration of high-density lipoprotein catabolism by endothelial lipase. Circulation.

[42] Adorni M.P., Ronda N., Bernini F., Zimetti F. (2021). High Density Lipoprotein Cholesterol Efflux Capacity and Atherosclerosis in Cardiovascular Disease: Pathophysiological Aspects and Pharmacological Perspectives. Cells.

[43] Le Lay S., Robichon C., Le Liepvre X., Dagher G., Ferre P., Dugail I. (2003). Regulation of ABCA1 expression and cholesterol efflux during adipose differentiation of 3T3-L1 cells. J. Lipid Res..

[44] Yu B.L., Zhao S.P., Hu J.R. (2010). Cholesterol imbalance in adipocytes: A possible mechanism of adipocytes dysfunction in obesity. Obes. Rev..

[45] de Haan W., Bhattacharjee A., Ruddle P., Kang M.H., Hayden M.R. (2014). ABCA1 in adipocytes regulates adipose tissue lipid content, glucose tolerance, and insulin sensitivity. J. Lipid Res..

[46] Jiang X.C., Moulin P., Quinet E., Goldberg I.J., Yacoub L.K., Agellon L.B., Compton D., Schnitzer-Polokoff R., Tall A.R. (1991). Mammalian adipose tissue and muscle are major sources of lipid transfer protein mRNA. J. Biol. Chem..

[47] Arai T., Yamashita S., Hirano K., Sakai N., Kotani K., Fujioka S., Nozaki S., Keno Y., Yamane M., Shinohara E. (1994). Increased plasma cholesteryl ester transfer protein in obese subjects. A possible mechanism for the reduction of serum HDL cholesterol levels in obesity. Arterioscler. Thromb..

[48] Denimal D., Monier S., Brindisi M.C., Petit J.M., Bouillet B., Nguyen A., Demizieux L., Simoneau I., Pais de Barros J.P., Verges B. (2017). Impairment of the Ability of HDL From Patients With Metabolic Syndrome but Without Diabetes Mellitus to Activate eNOS: Correction by S1P Enrichment. Arterioscler. Thromb. Vasc. Biol..

[49] Hui N., Barter P.J., Ong K.L., Rye K.A. (2019). Altered HDL metabolism in metabolic disorders: Insights into the therapeutic potential of HDL. Clin. Sci..

[50] Persegol L., Verges B., Gambert P., Duvillard L. (2007). Inability of HDL from abdominally obese subjects to counteract the inhibitory effect of oxidized LDL on vasorelaxation. J. Lipid Res..

[51] Sasahara T., Nestel P., Fidge N., Sviridov D. (1998). Cholesterol transport between cells and high density lipoprotein subfractions from obese and lean subjects. J. Lipid Res..

[52] Carr M.C., Hokanson J.E., Zambon A., Deeb S.S., Barrett P.H., Purnell J.Q., Brunzell J.D. (2001). The contribution of intraabdominal fat to gender differences in hepatic lipase activity and low/high density lipoprotein heterogeneity. J. Clin. Endocrinol. Metab..

[53] Chatterjee C., Sparks D.L. (2011). Hepatic lipase, high density lipoproteins, and hypertriglyceridemia. Am. J. Pathol..

[54] Rashid S., Barrett P.H., Uffelman K.D., Watanabe T., Adeli K., Lewis G.F. (2002). Lipolytically modified triglyceride-enriched HDLs are rapidly cleared from the circulation. Arterioscler. Thromb. Vasc. Biol..

[55] Zhao Y., He X., Shi X., Huang C., Liu J., Zhou S., Heng C.K. (2010). Association between serum amyloid A and obesity: A meta-analysis and systematic review. Inflamm. Res..

[56] Nigro E., Scudiero O., Monaco M.L., Palmieri A., Mazzarella G., Costagliola C., Bianco A., Daniele A. (2014). New insight into adiponectin role in obesity and obesity-related diseases. Biomed. Res. Int..

[57] Ouchi N., Walsh K. (2007). Adiponectin as an anti-inflammatory factor. Clin. Chim. Acta.

[58] Stefan N., Stumvoll M. (2002). Adiponectin—Its role in metabolism and beyond. Horm. Metab. Res..

[59] Hu E., Liang P., Spiegelman B.M. (1996). AdipoQ is a novel adipose-specific gene dysregulated in obesity. J. Biol. Chem..

[60] Kadowaki T., Yamauchi T., Kubota N., Hara K., Ueki K., Tobe K. (2006). Adiponectin and adiponectin receptors in insulin resistance, diabetes, and the metabolic syndrome. J. Clin. Investig..

[61] Hotta K., Funahashi T., Arita Y., Takahashi M., Matsuda M., Okamoto Y., Iwahashi H., Kuriyama H., Ouchi N., Maeda K. (2000). Plasma concentrations of a novel, adipose-specific protein, adiponectin, in type 2 diabetic patients. Arterioscler. Thromb. Vasc. Biol..

[62] Hafiane A., Gasbarrino K., Daskalopoulou S.S. (2019). The role of adiponectin in cholesterol efflux and HDL biogenesis and metabolism. Metab. Clin. Exp..

[63] Christou G.A., Kiortsis D.N. (2013). Adiponectin and lipoprotein metabolism. Obes. Rev..

[64] Jorge-Galarza E., Medina-Urrutia A., Reyes-Barrera J., Torres-Tamayo M., Montano-Estrada L.F., Paez-Arenas A., Masso-Rojas F., Juarez-Rojas J.G. (2023). Adipose tissue dysfunction serum markers are associated with high density lipoprotein size and glycation in the early stages of type 2 diabetes. Lipids Health Dis..

[65] Matsuura F., Oku H., Koseki M., Sandoval J.C., Yuasa-Kawase M., Tsubakio-Yamamoto K., Masuda D., Maeda N., Tsujii K., Ishigami M. (2007). Adiponectin accelerates reverse cholesterol transport by increasing high density lipoprotein assembly in the liver. Biochem. Biophys. Res. Commun..

[66] Obradovic M., Sudar-Milovanovic E., Soskic S., Essack M., Arya S., Stewart A.J., Gojobori T., Isenovic E.R. (2021). Leptin and Obesity: Role and Clinical Implication. Front. Endocrinol..

[67] Wu D.M., Shen M.H., Chu N.F. (2001). Relationship between plasma leptin levels and lipid profiles among school children in Taiwan—The Taipei Children Heart Study. Eur. J. Epidemiol..

[68] Lundasen T., Liao W., Angelin B., Rudling M. (2003). Leptin induces the hepatic high density lipoprotein receptor scavenger receptor B type I (SR-BI) but not cholesterol 7alpha-hydroxylase (Cyp7a1) in leptin-deficient (ob/ob) mice. J. Biol. Chem..

[69] Genchi V.A., D’Oria R., Palma G., Caccioppoli C., Cignarelli A., Natalicchio A., Laviola L., Giorgino F., Perrini S. (2021). Impaired Leptin Signalling in Obesity: Is Leptin a New Thermolipokine?. Int. J. Mol. Sci..

[70] McGillicuddy F.C., Reilly M.P., Rader D.J. (2011). Adipose modulation of high-density lipoprotein cholesterol: Implications for obesity, high-density lipoprotein metabolism, and cardiovascular disease. Circulation.

[71] Juarez-Rojas J.G., Torre-Villalvazo I., Medina-Urrutia A.X., Reyes-Barrera J., Sainz-Escarrega V.H., Posadas-Romero C., Macias-Cruz A., Jorge-Galarza E. (2020). Participation of white adipose tissue dysfunction on circulating HDL cholesterol and HDL particle size in apparently healthy humans. Int. J. Obes..

[72] Dullaart R.P., de Vries R., Dallinga-Thie G.M., van Tol A., Sluiter W.J. (2007). Plasma cholesteryl ester transfer protein mass and phospholipid transfer protein activity are associated with leptin in type 2 diabetes mellitus. Biochim. Biophys. Acta.

[73] Bamba V., Rader D.J. (2007). Obesity and atherogenic dyslipidemia. Gastroenterology.

[74] Skurk T., Alberti-Huber C., Herder C., Hauner H. (2007). Relationship between adipocyte size and adipokine expression and secretion. J. Clin. Endocrinol. Metab..

[75] Haas M.J., Horani M., Mreyoud A., Plummer B., Wong N.C., Mooradian A.D. (2003). Suppression of apolipoprotein AI gene expression in HepG2 cells by TNF alpha and IL-1beta. Biochim. Biophys. Acta.

[76] Beers A., Haas M.J., Wong N.C., Mooradian A.D. (2006). Inhibition of apolipoprotein AI gene expression by tumor necrosis factor alpha: Roles for MEK/ERK and JNK signaling. Biochemistry.

[77] Camps J., Garcia-Heredia A., Rull A., Alonso-Villaverde C., Aragones G., Beltran-Debon R., Rodriguez-Gallego E., Joven J. (2012). PPARs in Regulation of Paraoxonases: Control of Oxidative Stress and Inflammation Pathways. PPAR Res..

[78] Ferretti G., Bacchetti T. (2012). Effect of dietary lipids on paraoxonase-1 activity and gene expression. Nutr. Metab. Cardiovasc. Dis..

[79] Deakin S.P., James R.W. (2004). Genetic and environmental factors modulating serum concentrations and activities of the antioxidant enzyme paraoxonase-1. Clin. Sci..

[80] Deakin S., Moren X., James R.W. (2007). HDL oxidation compromises its influence on paraoxonase-1 secretion and its capacity to modulate enzyme activity. Arterioscler. Thromb. Vasc. Biol..

[81] Yang R.Z., Lee M.J., Hu H., Pollin T.I., Ryan A.S., Nicklas B.J., Snitker S., Horenstein R.B., Hull K., Goldberg N.H. (2006). Acute-phase serum amyloid A: An inflammatory adipokine and potential link between obesity and its metabolic complications. PLoS Med..

[82] Jousilahti P., Salomaa V., Rasi V., Vahtera E., Palosuo T. (2001). The association of c-reactive protein, serum amyloid a and fibrinogen with prevalent coronary heart disease–baseline findings of the PAIS project. Atherosclerosis.

[83] Wroblewski J.M., Jahangiri A., Ji A., de Beer F.C., van der Westhuyzen D.R., Webb N.R. (2011). Nascent HDL formation by hepatocytes is reduced by the concerted action of serum amyloid A and endothelial lipase. J. Lipid Res..

[84] Sato M., Ohkawa R., Yoshimoto A., Yano K., Ichimura N., Nishimori M., Okubo S., Yatomi Y., Tozuka M. (2016). Effects of serum amyloid A on the structure and antioxidant ability of high-density lipoprotein. Biosci. Rep..

[85] Vaisar T., Tang C., Babenko I., Hutchins P., Wimberger J., Suffredini A.F., Heinecke J.W. (2015). Inflammatory remodeling of the HDL proteome impairs cholesterol efflux capacity. J. Lipid Res..

[86] Bacchetti T., Ferretti G., Carbone F., Ministrini S., Montecucco F., Jamialahmadi T., Sahebkar A. (2021). Dysfunctional High-density Lipoprotein: The Role of Myeloperoxidase and Paraoxonase-1. Curr. Med. Chem..

[87] Zheng L., Nukuna B., Brennan M.L., Sun M., Goormastic M., Settle M., Schmitt D., Fu X., Thomson L., Fox P.L. (2004). Apolipoprotein A-I is a selective target for myeloperoxidase-catalyzed oxidation and functional impairment in subjects with cardiovascular disease. J. Clin. Investig..

[88] Sladoje D.P., Kisic B., Miric D. (2017). The Monitoring of Protein Markers of Inflammation and Serum Lipid Concentration in Obese Subjects with Metabolic Syndrome. J. Med. Biochem..

[89] Heinecke J.W., Goldberg I.J. (2014). Myeloperoxidase: A therapeutic target for preventing insulin resistance and the metabolic sequelae of obesity?. Diabetes.

[90] Andrade V.L., Petruceli E., Belo V.A., Andrade-Fernandes C.M., Caetano Russi C.V., Bosco A.A., Tanus-Santos J.E., Sandrim V.C. (2012). Evaluation of plasmatic MMP-8, MMP-9, TIMP-1 and MPO levels in obese and lean women. Clin. Biochem..

[91] Gomez Garcia A., Rivera Rodriguez M., Gomez Alonso C., Rodriguez Ochoa D.Y., Alvarez Aguilar C. (2015). Myeloperoxidase is associated with insulin resistance and inflammation in overweight subjects with first-degree relatives with type 2 diabetes mellitus. Diabetes Metab. J..

[92] Vazquez E., Sethi A.A., Freeman L., Zalos G., Chaudhry H., Haser E., Aicher B.O., Aponte A., Gucek M., Kato G.J. (2012). High-density lipoprotein cholesterol efflux, nitration of apolipoprotein A-I, and endothelial function in obese women. Am. J. Cardiol..

[93] Szentpeteri A., Zsiros N., Varga V.E., Lorincz H., Katko M., Seres I., Fulop P., Paragh G., Harangi M. (2017). Paraoxonase-1 and myeloperoxidase correlate with vascular biomarkers in overweight patients with newly diagnosed untreated hyperlipidaemia. Vasa.

[94] Zsiros N., Koncsos P., Lorincz H., Seres I., Katko M., Szentpeteri A., Varga V.E., Fulop P., Paragh G., Harangi M. (2016). Paraoxonase-1 arylesterase activity is an independent predictor of myeloperoxidase levels in overweight patients with or without cardiovascular complications. Clin. Biochem..

[95] van Marken Lichtenbelt W.D., Vanhommerig J.W., Smulders N.M., Drossaerts J.M., Kemerink G.J., Bouvy N.D., Schrauwen P., Teule G.J. (2009). Cold-activated brown adipose tissue in healthy men. N. Engl. J. Med..

[96] Heeren J., Scheja L. (2018). Brown adipose tissue and lipid metabolism. Curr. Opin. Lipidol..

[97] Chen H.J., Meng T., Gao P.J., Ruan C.C. (2021). The Role of Brown Adipose Tissue Dysfunction in the Development of Cardiovascular Disease. Front. Endocrinol..

[98] Tabei S., Chamorro R., Meyhofer S.M., Wilms B. (2024). Metabolic Effects of Brown Adipose Tissue Activity Due to Cold Exposure in Humans: A Systematic Review and Meta-Analysis of RCTs and Non-RCTs. Biomedicines.

[99] Bays H.E., Kirkpatrick C., Maki K.C., Toth P.P., Morgan R.T., Tondt J., Christensen S.M., Dixon D., Jacobson T.A. (2024). Obesity, dyslipidemia, and cardiovascular disease: A joint expert review from the Obesity Medicine Association and the National Lipid Association 2024. Obes Pillars.

[100] Kirkpatrick C.F., Sikand G., Petersen K.S., Anderson C.A.M., Aspry K.E., Bolick J.P., Kris-Etherton P.M., Maki K.C. (2023). Nutrition interventions for adults with dyslipidemia: A Clinical Perspective from the National Lipid Association. J. Clin. Lipidol..

[101] Sikand G., Severson T. (2020). Top 10 dietary strategies for atherosclerotic cardiovascular risk reduction. Am. J. Prev. Cardiol..

[102] Bays H.E., Gonsahn-Bollie S., Younglove C., Wharton S. (2022). Obesity Pillars Roundtable: Body mass index and body composition in Black and Female individuals. Race-relevant or racist? Sex-relevant or sexist?. Obes. Pillars.

[103] Petrovic A., Jovicic S., Dodevska M., Djordjevic B., Milinkovic N., Ivanovic N.D. (2024). Effects of Specially Designed Energy-Restricted Diet on Anthropometric Parameters and Cardiometabolic Risk in Overweight and Obese Adults: Pilot Study. Nutrients.

[104] Holowko J., Michalczyk M.M., Zajac A., Czerwinska-Rogowska M., Ryterska K., Banaszczak M., Jakubczyk K., Stachowska E. (2019). Six Weeks of Calorie Restriction Improves Body Composition and Lipid Profile in Obese and Overweight Former Athletes. Nutrients.

[105] Cioffi I., Evangelista A., Ponzo V., Ciccone G., Soldati L., Santarpia L., Contaldo F., Pasanisi F., Ghigo E., Bo S. (2018). Intermittent versus continuous energy restriction on weight loss and cardiometabolic outcomes: A systematic review and meta-analysis of randomized controlled trials. J. Transl. Med..

[106] Stadler J.T., Marsche G. (2021). Dietary Strategies to Improve Cardiovascular Health: Focus on Increasing High-Density Lipoprotein Functionality. Front. Nutr..

[107] Lopez-Domenech S., Martinez-Herrera M., Abad-Jimenez Z., Morillas C., Escribano-Lopez I., Diaz-Morales N., Banuls C., Victor V.M., Rocha M. (2019). Dietary weight loss intervention improves subclinical atherosclerosis and oxidative stress markers in leukocytes of obese humans. Int. J. Obes..

[108] Kanikowska D., Kanikowska A., Swora-Cwynar E., Grzymislawski M., Sato M., Breborowicz A., Witowski J., Korybalska K. (2021). Moderate Caloric Restriction Partially Improved Oxidative Stress Markers in Obese Humans. Antioxidants.

[109] Montefusco L., D’Addio F., Loretelli C., Ben Nasr M., Garziano M., Rossi A., Pastore I., Plebani L., Lunati M.E., Bolla A.M. (2021). Anti-inflammatory effects of diet and caloric restriction in metabolic syndrome. J. Endocrinol. Investig..

[110] Poitou C., Viguerie N., Cancello R., De Matteis R., Cinti S., Stich V., Coussieu C., Gauthier E., Courtine M., Zucker J.D. (2005). Serum amyloid A: Production by human white adipocyte and regulation by obesity and nutrition. Diabetologia.

[111] Yang W.S., Lee W.J., Funahashi T., Tanaka S., Matsuzawa Y., Chao C.L., Chen C.L., Tai T.Y., Chuang L.M. (2001). Weight reduction increases plasma levels of an adipose-derived anti-inflammatory protein, adiponectin. J. Clin. Endocrinol. Metab..

[112] Bosch-Sierra N., Grau-Del Valle C., Hermenejildo J., Hermo-Argibay A., Salazar J.D., Garrido M., Navajas-Porras B., Saez G., Morillas C., Banuls C. (2024). The Impact of Weight Loss on Inflammation, Oxidative Stress, and Mitochondrial Function in Subjects with Obesity. Antioxidants.

[113] Dominguez L.J., Veronese N., Di Bella G., Cusumano C., Parisi A., Tagliaferri F., Ciriminna S., Barbagallo M. (2023). Mediterranean diet in the management and prevention of obesity. Exp. Gerontol..

[114] Mathew A.V., Li L., Byun J., Guo Y., Michailidis G., Jaiswal M., Chen Y.E., Pop-Busui R., Pennathur S. (2018). Therapeutic Lifestyle Changes Improve HDL Function by Inhibiting Myeloperoxidase-Mediated Oxidation in Patients With Metabolic Syndrome. Diabetes Care.

[115] El Oirdi M. (2024). Harnessing the Power of Polyphenols: A New Frontier in Disease Prevention and Therapy. Pharmaceuticals.

[116] Bacchetti T., Turco I., Urbano A., Morresi C., Ferretti G. (2019). Relationship of fruit and vegetable intake to dietary antioxidant capacity and markers of oxidative stress: A sex-related study. Nutrition.

[117] Nani A., Murtaza B., Sayed Khan A., Khan N.A., Hichami A. (2021). Antioxidant and Anti-Inflammatory Potential of Polyphenols Contained in Mediterranean Diet in Obesity: Molecular Mechanisms. Molecules.

[118] Siriwardhana N., Kalupahana N.S., Cekanova M., LeMieux M., Greer B., Moustaid-Moussa N. (2013). Modulation of adipose tissue inflammation by bioactive food compounds. J. Nutr. Biochem..

[119] Wang S., Moustaid-Moussa N., Chen L., Mo H., Shastri A., Su R., Bapat P., Kwun I., Shen C.L. (2014). Novel insights of dietary polyphenols and obesity. J. Nutr. Biochem..

[120] Yahfoufi N., Alsadi N., Jambi M., Matar C. (2018). The Immunomodulatory and Anti-Inflammatory Role of Polyphenols. Nutrients.

[121] Lee Y.M., Yoon Y., Yoon H., Park H.M., Song S., Yeum K.J. (2017). Dietary Anthocyanins against Obesity and Inflammation. Nutrients.

[122] Esfahani A., Wong J.M., Truan J., Villa C.R., Mirrahimi A., Srichaikul K., Kendall C.W. (2011). Health effects of mixed fruit and vegetable concentrates: A systematic review of the clinical interventions. J. Am. Coll. Nutr..

[123] Martemucci G., Khalil M., Di Luca A., Abdallah H., D’Alessandro A.G. (2024). Comprehensive Strategies for Metabolic Syndrome: How Nutrition, Dietary Polyphenols, Physical Activity, and Lifestyle Modifications Address Diabesity, Cardiovascular Diseases, and Neurodegenerative Conditions. Metabolites.

[124] Silveira J.Q., Dourado G.K., Cesar T.B. (2015). Red-fleshed sweet orange juice improves the risk factors for metabolic syndrome. Int. J. Food Sci. Nutr..

[125] Zunino S.J., Parelman M.A., Freytag T.L., Stephensen C.B., Kelley D.S., Mackey B.E., Woodhouse L.R., Bonnel E.L. (2012). Effects of dietary strawberry powder on blood lipids and inflammatory markers in obese human subjects. Br. J. Nutr..

[126] Azzini E., Venneria E., Ciarapica D., Foddai M.S., Intorre F., Zaccaria M., Maiani F., Palomba L., Barnaba L., Tubili C. (2017). Effect of Red Orange Juice Consumption on Body Composition and Nutritional Status in Overweight/Obese Female: A Pilot Study. Oxid. Med. Cell. Longev..

[127] Wang Y., Snel M., Jonker J.T., Hammer S., Lamb H.J., de Roos A., Meinders A.E., Pijl H., Romijn J.A., Smit J.W. (2011). Prolonged caloric restriction in obese patients with type 2 diabetes mellitus decreases plasma CETP and increases apolipoprotein AI levels without improving the cholesterol efflux properties of HDL. Diabetes Care.

[128] Liang K.W., Lee W.J., Lee I.T., Lee W.L., Lin S.Y., Hsu S.L., Wan C.J., Yu C.Y., Tsai I.C., Fu C.P. (2011). Persistent elevation of paraoxonase-1 specific enzyme activity after weight reduction in obese non-diabetic men with metabolic syndrome. Clin. Chim. Acta.

[129] Kotani K., Sakane N., Sano Y., Tsuzaki K., Matsuoka Y., Egawa K., Yoshimura M., Horikawa C., Kitagawa Y., Kiso Y. (2009). Changes on the physiological lactonase activity of serum paraoxonase 1 by a diet intervention for weight loss in healthy overweight and obese women. J. Clin. Biochem. Nutr..

[130] Amiot M.J., Riva C., Vinet A. (2016). Effects of dietary polyphenols on metabolic syndrome features in humans: A systematic review. Obes. Rev..

[131] Millar C.L., Duclos Q., Blesso C.N. (2017). Effects of Dietary Flavonoids on Reverse Cholesterol Transport, HDL Metabolism, and HDL Function. Adv. Nutr..

[132] Castro-Barquero S., Tresserra-Rimbau A., Vitelli-Storelli F., Domenech M., Salas-Salvado J., Martin-Sanchez V., Rubin-Garcia M., Buil-Cosiales P., Corella D., Fito M. (2020). Dietary Polyphenol Intake is Associated with HDL-Cholesterol and A Better Profile of other Components of the Metabolic Syndrome: A PREDIMED-Plus Sub-Study. Nutrients.

[133] Wisnuwardani R.W., De Henauw S., Androutsos O., Forsner M., Gottrand F., Huybrechts I., Knaze V., Kersting M., Le Donne C., Marcos A. (2019). Estimated dietary intake of polyphenols in European adolescents: The HELENA study. Eur. J. Nutr..

[134] Kawser Hossain M., Abdal Dayem A., Han J., Yin Y., Kim K., Kumar Saha S., Yang G.M., Choi H.Y., Cho S.G. (2016). Molecular Mechanisms of the Anti-Obesity and Anti-Diabetic Properties of Flavonoids. Int. J. Mol. Sci..

[135] Curtis P.J., van der Velpen V., Berends L., Jennings A., Feelisch M., Umpleby A.M., Evans M., Fernandez B.O., Meiss M.S., Minnion M. (2019). Blueberries improve biomarkers of cardiometabolic function in participants with metabolic syndrome-results from a 6-month, double-blind, randomized controlled trial. Am. J. Clin. Nutr..

[136] Macedo A.P.A., Goncalves M.D.S., Barreto Medeiros J.M., David J.M., Villarreal C.F., Macambira S.G., Soares M.B.P., Couto R.D. (2022). Potential therapeutic effects of green tea on obese lipid profile—A systematic review. Nutr. Health.

[137] Chatree S., Sitticharoon C., Maikaew P., Pongwattanapakin K., Keadkraichaiwat I., Churintaraphan M., Sripong C., Sririwichitchai R., Tapechum S. (2021). Epigallocatechin gallate decreases plasma triglyceride, blood pressure, and serum kisspeptin in obese human subjects. Exp. Biol. Med..

[138] Li A., Wang Q., Li P., Zhao N., Liang Z. (2024). Effects of green tea on lipid profile in overweight and obese women. Int. J. Vitam. Nutr. Res..

[139] Jeffery R.W., Drewnowski A., Epstein L.H., Stunkard A.J., Wilson G.T., Wing R.R., Hill D.R. (2000). Long-term maintenance of weight loss: Current status. Health Psychol..

